# Role of Notch1 in the arterial specification and angiogenic potential of mouse embryonic stem cell-derived endothelial cells

**DOI:** 10.1186/s13287-018-0945-7

**Published:** 2018-07-18

**Authors:** Jae Kyung Park, Tae Wook Lee, Eun Kyoung Do, Hye Ji Moon, Jae Ho Kim

**Affiliations:** 1 Department of Physiology, School of Medicine, Pusan National University, Yangsan, 50612 Gyeongsangnam-do Republic of Korea; 2 Research Institute of Convergence Biomedical Science and Technology, Pusan National University Yangsan Hospital, Yangsan, 50612 Republic of Korea

**Keywords:** Notch1, Mouse embryonic stem cell, Endothelial cell, Angiogenesis, Peripheral artery disease, Hindlimb ischemia

## Abstract

**Background:**

Endothelial cells have been shown to mediate angiogenesis in ischemic injury sites and contribute to the repair of damaged tissues. However, the treatment of ischemic disease requires a significant number of endothelial cells, which are difficult to isolate from patients. Embryonic stem cells have been considered a potential source of therapeutic cells due to their unlimited self-renewal and pluripotent properties. With regard to vascular development, Notch1 has been established as a key regulator of the specification of arterial endothelial cells.

**Methods:**

Using a doxycycline-induced expression system of the intracellular domain of Notch1, we explored the role of Notch1 in the differentiation of embryonic stem cells to arterial endothelial cells. The therapeutic effect of the arterial endothelial cells was investigated in a murine hindlimb ischemia model. The blood perfusion rate in the ischemic limb was determined by laser Doppler perfusion imaging, and vasculogenesis was quantified using immunocytochemistry.

**Results:**

Induced expression of the intracellular domain of Notch1 increased the levels of endothelial markers, such as CD31 and VE-cadherin, in differentiated endothelial cells. Induction of intracellular domain of Notch1 stimulated expression of the arterial-type endothelial cell markers (Nrp1 and Ephrin B2), but not the venous-type endothelial cell markers (Nrp2 and Coup-TFII). In addition, overexpression of intracellular domain of Notch1 resulted in increased expression of CXCR4, a chemokine receptor involved in vascular development. Induction of intracellular domain of Notch1 increased endothelial tube formation and migration of differentiated endothelial cells. Intramuscular administration of Notch1-induced arterial endothelial cells was more effective than administration of the control endothelial cells in restoring the blood flow in an ischemic hindlimb mouse model. Transplantation of Notch1-induced arterial endothelial cells augmented the number of blood vessels and incorporation of endothelial cells into newly formed blood vessels.

**Conclusions:**

These results suggest that Notch1 promotes endothelial maturation and arterial specification during the differentiation of embryonic stem cells to endothelial cells and increases the angiogenic potential of endothelial cells.

**Electronic supplementary material:**

The online version of this article (10.1186/s13287-018-0945-7) contains supplementary material, which is available to authorized users.

## Background

Ischemia is a major health care issue in the aging population [1]. Ischemia is a disease caused by accumulation of blood clots inside the blood vessels or by pressure exerted on blood vessels due to swelling from external injuries, resulting in decreased blood flow. One way to treat ischemic diseases is to form new blood vessels. Angiogenesis refers to the formation of blood vessels that sprout from pre-existing blood vessels [2]. Angiogenesis involves vascular endothelial cells (ECs), which are essential for maintaining vascular structure [3]. Cell therapy in ischemic diseases requires a number of ECs; however, it is difficult to acquire and isolate ECs from patients. Additionally, protocols for isolating ECs have typically low efficiency.

Embryonic stem cells (ESCs) and induced pluripotent stem cells have been suggested to be the solution to this problem. ESCs possess self-renewal potential and can be differentiated into all phenotypes; therefore, ESCs are highly useful for cell therapy [4]. In order to differentiate ESCs into ECs, cells must be differentiated into mesodermal progenitor cells that can give rise to ECs and mural cells [5]. Vascular endothelial growth factor receptor 2, also called Flk1, is a marker for the lateral mesoderm [6]. Flk1-expressing (Flk1^+^) cells derived from ESCs differentiate ECs and blood cells in vitro [7, 8]. Importantly, transplantation of ESC-derived ECs promoted in-vivo angiogenesis in the embryo [9]. These results suggest that ESC-derived ECs can be used for the treatment of ischemic diseases.

Notch signaling was activated via interaction with Notch ligands, such as Delta-like 4, Jagged1, and Jagged2, from neighboring cells [10]. When Notch signaling is activated, the Notch receptor is cleaved by gamma-secretase to release intracellular domain of Notch1 (ICN1), which then translocates to the nucleus and activates the expression of Notch downstream genes. Notch signaling is important in determining artery and vein ECs as well as in the differentiation of mesodermal cells into mesodermal angioblasts [11]. In developing ECs, Hey1 and Hey2 (target transcription factors for Notch signaling) have been reported to stimulate arterial-type EC differentiation and suppress venous-type EC differentiation [12]. Alternatively, with regard to the differentiation of venous-type ECs, COUF-TFII has been reported to induce differentiation of venous-type ECs through the suppression of Notch signaling [13]. However, the molecular mechanism by which Notch1 regulates differentiation of ESCs to arterial ECs has yet to be identified.

In this study, we evaluated the effects of Notch1 signaling on the differentiation of ESCs to ECs using doxycycline (Dox)-inducible intracellular domain of Notch1 (iICN1) mouse ESCs. In addition, we explored the effect of ESC-derived atrial ECs on the repair of hindlimb ischemia. Results of the present study suggest that Notch1 promotes arterial specification of ECs derived from ESCs and enhances ischemic tissue repair by stimulating the angiogenic potential of the differentiated ECs.

## Methods

### Materials

Dulbecco’s Modified Eagle Medium (DMEM), nonessential amino acids, Glutamax-1, penicillin–streptomycin, β-mercaptoethanol, Iscove’s Modified Dulbecco’s Medium (IMDM), Ham’s F12 medium, N-2 and B-27 Supplements (without vitamin A), bovine serum albumin, Accutase cell detachment solution, and Alexa Fluor® 488-conjugated anti-VE-cadherin antibody (53-1441-82) were purchased from Thermo Fisher Scientific (Waltham, MA, USA). Basic fibroblast growth factor, bone morphogenetic protein-4, vascular endothelial growth factor, anti-Neuropilin-2 antibody (AF567), and anti-Ephrin B2 (AF496) antibody were purchased from R&D Systems (Minneapolis, MN, USA). Anti-Phycoerythrin (PE) microbeads (130-048-801) and an LS separation column (130-42-401) were purchased from Miltenyi Biotec (Auburn, CA, USA). Antibodies against OCT4 (sc-8628), Flk1 (sc-505), and SSEA-1 (sc-21,702) were purchased from Santa Cruz Biotechnology (Dallas, TX, USA). Antibodies against CD31 (ab28364), Neuropilin-1 (ab81321), Coup-TFII (ab41859), and VE-cadherin (ab33168) were purchased from Abcam plc. (Boston, MA, USA). Anti-Notch1 (#4380) and anti-Nanog (#8822) antibodies was purchased from Cell Signaling Technology (Danvers, MA, USA). PE-conjugated anti-CXCR4 antibody (551966) was purchased from BD Biosciences (San Jose, CA, USA). Alexa Fluor® 647-conjugated anti-CD31 antibody (102416) was purchased from BioLegend (San Diego, CA, USA). Hank’s Balanced Salt Solution (HBSS), trypsin, Mitomycin-C, ascorbic acid, 1-thioglycerol, and other unlisted reagents were purchased from Sigma-Aldrich (St. Louis, MO, USA).

### Cell culture

D3 and Dox–iICN1 mouse ESCs [14] were cultured on mitomycin-C-treated mouse embryonic fibroblasts (MEFs) in ESC culture media consisting of DMEM supplemented with 1× nonessential amino acids, 1× Glutamax-1, 15% FBS, 1× penicillin–streptomycin, 0.1 mM β-mercaptoethanol, and 1000 U/ml leukemia inhibitory factor. MEFs were isolated from 12.5-day-old embryos in pregnant C3H mice and maintained in MEF medium (DMEM with high glucose, 1× Glutamax-1, 10% FBS, and 1× penicillin–streptomycin), as described previously [15]. Protocols for the differentiation of mouse ESCs into ECs have also been described previously [16]. To initiate differentiation, the MEF-free preconditioned cells were dissociated with trypsin/ethylenediaminetetraacetic acid (EDTA) solution and seeded on 1 μg/cm^2^ type IV collagen (#354233; Corning, NY, USA)-coated dishes at a density of 35,000/35-mm dish. The cells were incubated with serum-free differentiation medium consisting of 75% IMDM, 25% Ham’s F12 medium, N-2 and B-27 Supplements (without vitamin A), 0.05% bovine serum albumin (all from Thermo Fisher Scientific), 0.45 mM 1-thioglycerol, 0.5 mM ascorbic acid, 2 ng/ml human bone morphogenetic protein-4, 50 ng/ml human vascular endothelial growth factor 165, and 10 ng/ml human basic fibroblast growth factor. On day 5 after incubation with differentiation medium, Flk1-positive mesodermal progenitor cells were isolated by magnetic-activated cell sorting. After cell sorting, the FLk1-positive cells were treated with 0.5 mg/ml doxycycline for the indicated time periods.

### Magnetic-activated cell sorting and fluorescence-activated cell sorting

For magnetic-activated cell sorting, the cells were digested with Accutase cell detachment solution. The dissociated cells were subsequently washed with HBSS buffer, centrifuged, and resuspended in 90 μl IMDM supplemented with 2% FBS plus 10 μl PE-conjugated anti-Flk1 antibody for 10 min at 4 °C, and then with 80 μl IMDM supplemented with 2% FBS plus 20 μl anti-PE microbeads for another 15 min at 4 °C. The cells were then resuspended with 0.5 ml IMDM supplemented with 2% FBS and passed through an LS separation column. After extensive washing, Flk1^+^ cells were collected from the LS column by flushing with 5 ml IMDM containing 2% FBS. The phenotypes of Flk1^+^ cells were analyzed on a FACS Canto II system (BD Biosciences).

### Flow cytometry analysis

To characterize the surface antigen on iICN1-ECs (iICN1-differentiated ECs), we performed fluorescence-activated cell sorter (FACS) analysis. Sorted cells were removed from the dish and resuspended in cold wash buffer (0.2% FBS in HBSS). The cells were incubated with PE-conjugated anti-CXCR4, Alexa Fluor® 488-conjugated anti-VE-cadherin, or Alexa Fluor® 647-conjugated anti-CD31 antibodies in the dark for 10 min at 4 °C, and then washed and resuspended in the wash buffer. Isotype-matched IgG antibodies (BD Pharmingen) were used as negative controls. Analysis of fluorescence intensity of the stained cells was performed using the FACS Canto II (BD Biosciences). Ten thousand events were acquired on the FACS Canto II flow cytometer (BD Biosciences), and data analysis was performed using the CellQuest software (BD Biosciences).

### Quantitative real time-polymerase chain reaction (qRT-PCR)

For RT-PCR analysis, 2 μg aliquots of each RNA were subjected to cDNA synthesis using 200 U of M-MLV reverse transcriptase and 0.5 μg of oligo(dT)15 primer (C-1101; Promega, Madison, WI, USA). qRT-PCR was performed on an ABI 7500 (Applied Biosystems) sequence detection system with SYBR Green PCR Master Mix (ABS-4309155; Applied Biosystems) according to the manufacturer’s instructions. Experiments were performed in triplicate, and the data were normalized to GAPDH mRNA expression. Data were analyzed using the Δ(ΔCT) method and normalized to GAPDH. The primer sequences are presented in Additional file 1: Table S1.

### Western blotting analysis

To prepare the cell extracts, mouse ESCs were provided with the appropriate conditions, washed with ice-cold phosphate-buffered saline (PBS), and then lysed in lysis buffer (20 mM Tris–HCl, 1 mM EGTA, 1 mM EDTA, 10 mM NaCl, 0.5 mM phenylmethylsulfonyl fluoride, 1 mM Na_3_VO_4_, 30 mM sodium pyrophosphate, 25 mM β-glycerol phosphate, 1% Triton X-100, pH 7.4). Lysates were resolved using sodium dodecyl sulfate polyacrylamide gel electrophoresis (SDS-PAGE), transferred onto a nitrocellulose membrane, and stained with 0.1% Ponceau S solution (P3504; Sigma-Aldrich). After blocking with 5% nonfat milk, the membranes were immunoblotted with various antibodies, and bound antibodies were visualized with horseradish peroxidase-conjugated secondary antibodies using the enhanced chemiluminescence western blotting system (GE Healthcare Life Sciences, Pittsburgh, PA, USA).

### Tube formation

In order to determine the tube-forming ability of the iICN1-ECs, growth factor-reduced Matrigel (354,230; BD Biosciences) was added to 48-well culture plates and polymerized for 30 min at 37 °C. iICN1-ECs (4 × 10^4^ cells) were seeded on Matrigel-coated plates and cultured in serum-free medium with 10 ng/ml vascular endothelial growth factor. After incubation of the cells at 37 °C and 5% CO_2_ for 24 h, they were gently washed with PBS and then incubated with 2 μM Calcein AM (Thermo Fisher Scientific) for 30 min. The Calcein AM was washed with PBS, and tube formation was detected by an inverted fluorescence microscopy (DM IRB; Leica Microsystems, Solms, Germany). The tube networks were quantified by measuring the tube length in four random microscope fields using ImageJ software (https://imagej.nih.gov/ij/).

### Cell migration assay

Chemotactic migration of iICN1-ECs was assayed using a disposable 96-well chemotaxis chamber. Transwell migration assays were performed according to the manufacturer’s instructions. Cells (5 × 10^3^) in 50 μl of serum-free medium were seeded onto the top of each membrane well. Serum-free medium (320 μl) was added to the lower chamber well. Following incubation for 12 h at 37 °C, noninvasive cells were removed from the membrane. Cells that invaded the membrane were fixed with 4% paraformaldehyde, stained with 0.1% Hoechst, and counted under the florescence microscope. The number of cells that had migrated to the lower surface of each filter was determined by counting the cells in four locations under microscopy at ×100 magnification.

### Hindlimb ischemia and blood flow measurement

BALB/C or BALB/CA-nu/nu (male, age 6–9 weeks, weighing 22–24 g) were anesthetized with an intraperitoneal injection of 400 mg/kg 2,2,2-tribromoethanol (Avertin; Sigma-Aldrich) for operative resection of one femoral artery and laser Doppler perfusion imaging (LDPI). The femoral artery was excised from its proximal origin as a branch of the external iliac artery to the distal point where it bifurcated into the saphenous and popliteal arteries. After arterial ligation, ischemic hindlimbs received injection with HBSS or iICN1-ECs (1 × 10^6^ cells in 60 μl HBSS) into three sites (20 μl/each site) of the muscle in the medial thigh three times per week. Perfusion of the ischemic and nonischemic limb was calculated based on colored histogram pixels. Red and blue indicated high and low perfusion, respectively. Blood perfusion is expressed as the LDPI index, which represents the ratio of ischemic versus nonischemic limb blood flow. A ratio of 1 before surgery indicates equivalent blood perfusion in both legs.

### Immunofluorescent staining and in-vivo tracking of transplanted cells

For immunofluorescence staining, mouse ESCs were fixed in 4% paraformaldehyde in PBS for 20 min, washed twice with PBS, and blocked with 1% FBS in PBS for 30 min. The fixed specimens were incubated with primary antibodies against OCT4 or SSEA-1 for 1 h. For immunostaining analysis of hindlimb tissues, the tissue specimens were embedded in paraffin. Six-micrometer paraffin sections were immunostained with rabbit anti-α-SMA or rat anti-CD31 antibodies. The specimens were then incubated with Alexa Fluor 488 or Alexa Fluor 568 conjugated secondary antibodies (1:1000; Life Technologies), followed by washing with PBS and mounting in VECTASHIED medium (Vector Laboratories, Burlingame, CA, USA) with 4′,6-diamidino-2-phenylindole (DAPI) for visualization of the nuclei. Stained sections were visualized using laser scanning confocal microscopy (FluoView FV1000; Olympus Corp., Tokyo, Japan), and blood vessel densities were assessed by counting the number of CD31-positive or α-SMA-positive blood vessels in three serial sections per ischemic tissue.

For in-vivo tracking of transplanted cells, iICN1-ECs were labeled with the CellTracker Green CMFDA (Thermo Fisher Scientific) according to the manufacturer’s instructions. The CMFDA-labeled iICN1-ECs (1 × 10^6^ cells in 60 μl HBSS) were injected into three sites (20 μl/each site) of the muscle. After 1 week, the mice were sacrificed and hindlimb tissue samples were fixed with 4% paraformaldehyde overnight and embedded in paraffin. Six-micrometer paraffin sections were stained with antibodies against CD31 or α-SMA, followed by mounting with VECTASHIED medium with DAPI (Vector Laboratories). The numbers of blood vessels expressing CD31 or α-SMA positive for CMFDA fluorescent dye were quantified in three serial sections, five high-power fields per section.

### Statistical analysis

Results of multiple observations are presented as the mean ± standard deviation (SD). Intergroup analysis was conducted using Student’s *t* test to compare differences between two groups. For analysis of multivariate data, group differences were assessed using one-way analysis of variance (ANOVA), followed by Scheffe’s post-hoc test. Statistical significance was indicated by *p* < 0.05.

## Results

### Characterization of iICN1 mouse ESCs

**Fig. 1:**
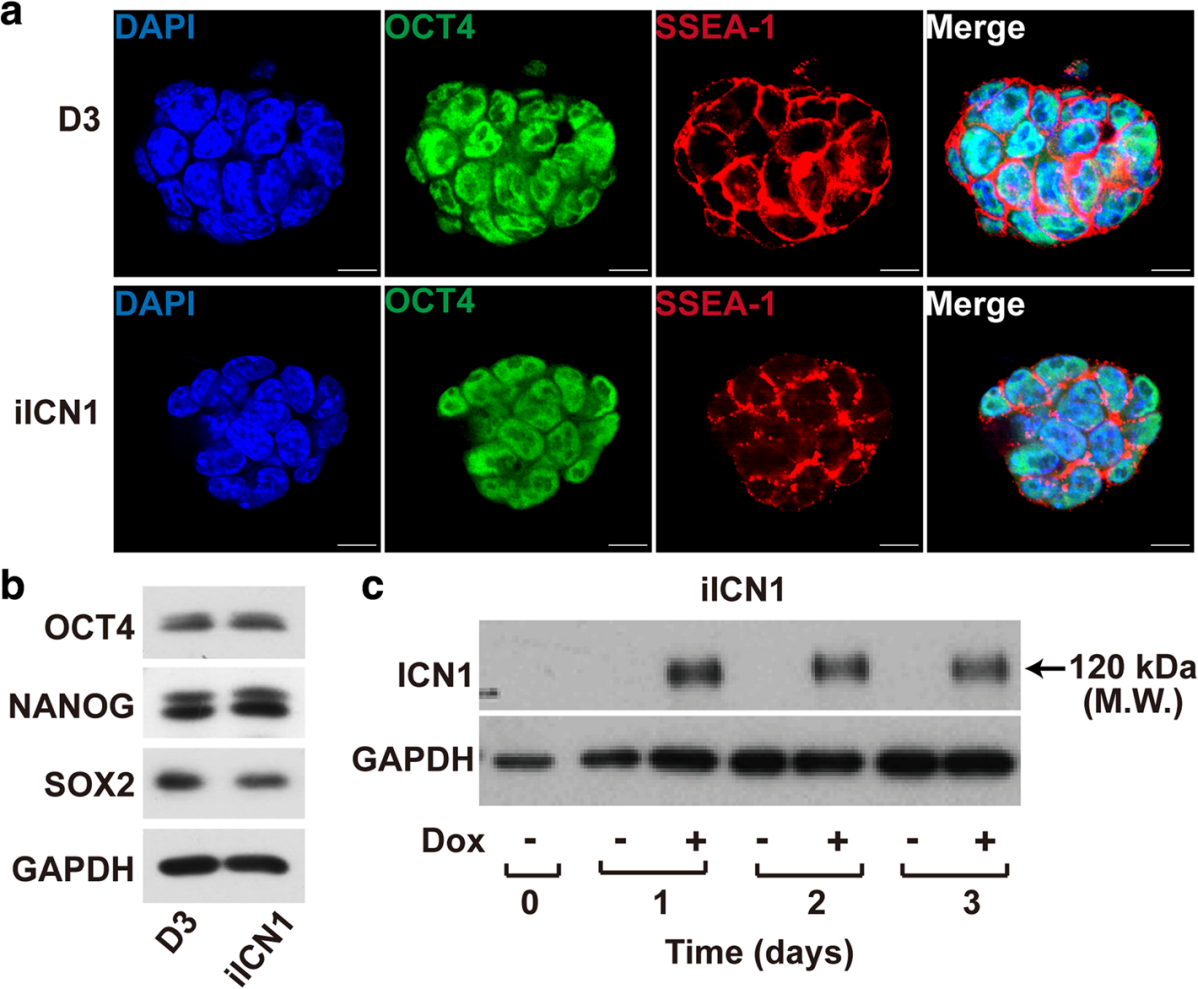
Similar expression of pluripotency markers in D3 and iICN1 ESCs. **a** Confocal immunofluorescence micrographs show expression of OCT4 (green), SSEA-1 (red), DAPI (blue), and merged images in D3 (upper panels) and iICN1 (lower panels) ESCs. Scale bars: 10 μm. **b** Western blot analysis of D3 and iICN1 ESCs with pluripotency markers (OCT4, NANOG, and SOX2) and GAPDH. **c** Expression level of ICN1 in iICN1 ESCs with (+) or without (−) Dox treatment for indicated time periods. Estimated molecular weight of ICN1 protein band indicated. Representative data from three independent experiments. DAPI 4′,6-diamidino-2-phenylindole, Dox doxycycline, GAPDH glyceraldehyde 3-phosphate dehydrogenase, iICN1 inducible intracellular domain of Notch1, M.W. molecular weight, SSEA-1 stage-specific embryonic antigen 1, SOX-2 sex determining region Y-box 2

iICN1 ESCs were morphologically similar to D3 ESCs. Immunocytochemistry analysis confirmed that both iICN1 and D3 ESCs expressed OCT4 and SSEA-1 (Fig. 1a). Western blotting data showed that iICN1 ESCs expressed specific pluripotency markers such as OCT4, SOX2, and NANOG, similar to D3 ESCs (Fig. 1b). We next tested whether the intracellular domain of Notch1 (ICN1) could be induced in iICN1 ESCs in a Dox-dependent manner. iICN1 ESCs were treated with 0.5 mg/ml doxycycline for the indicated time. The results showed that ICN1 expression was highly induced after exposure to Dox for 1 day (Fig. 1c).

### Notch1 contributes to the differentiation of mouse ESCs into ECs

**Fig. 2:**
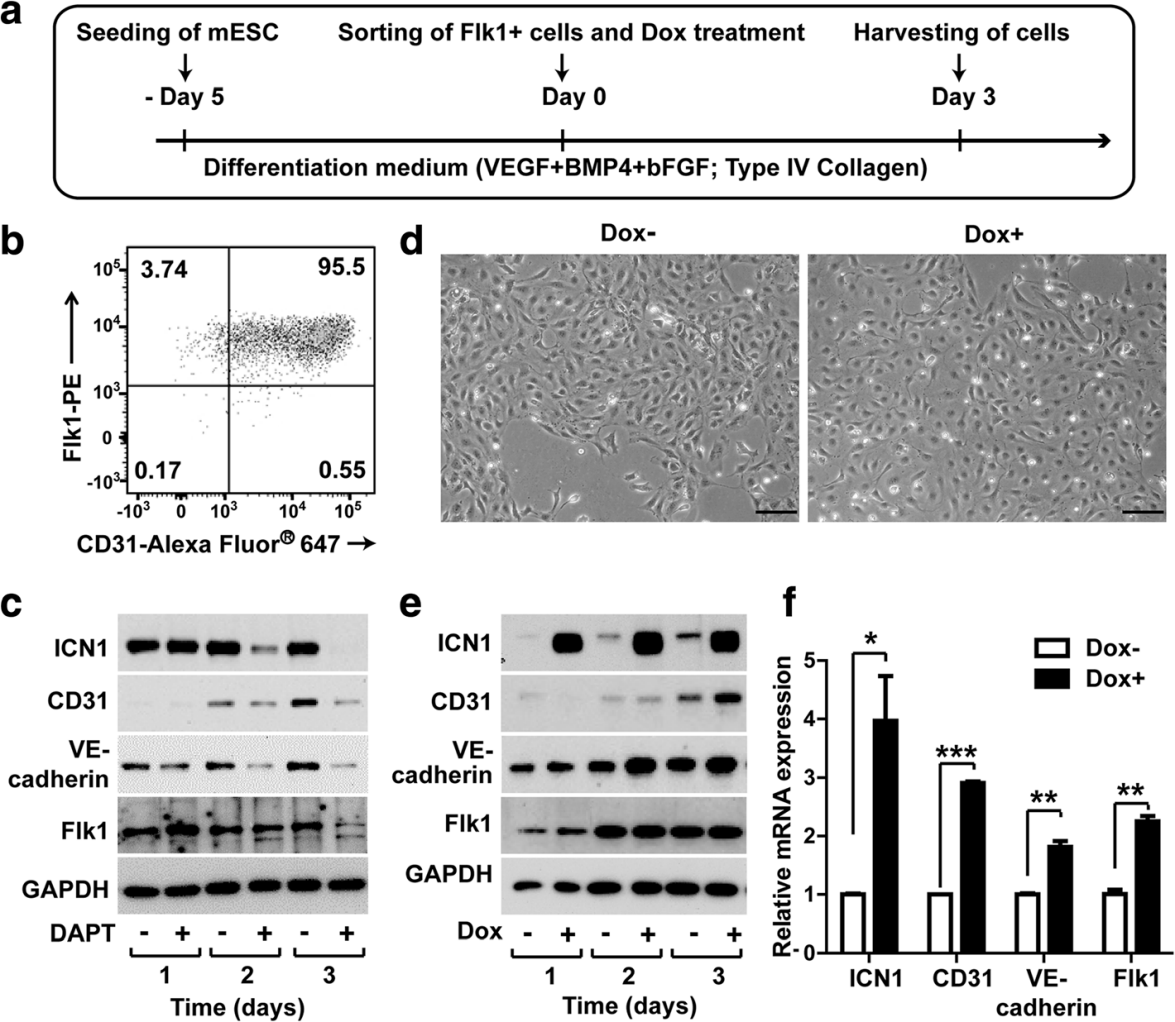
Effects of Notch1 activation on differentiation of ESC-derived ECs. **a** Timeline for differentiation of iICN1 ESCs into endothelial cells. **b** FACS analysis of Flk1-positive cells isolated by magnetic-activated cell sorting. Double staining of cells with PE-conjugated anti-Flk1 and Alexa Fluor® 647 anti-CD31 antibodies. Numbers denote percent of cells per quadrant. **c** Effects of γ-secretase inhibitor DAPT on endothelial differentiation of Flk1-positive iICN1-ECs. Flk1-positive cells treated 10 μM DAPT for indicated times, and expression levels of endothelial markers (Flk1, CD31, and VE-cadherin), ICN1, and GAPDH determined by western blotting analysis. **d** Representative photographs of iICN1-ECs after treatment with or without Dox for 3 days. Scale bars: 100 μm. **e** Effect of ICN1 induction on expression of endothelial markers in Flk1-positive cells. Flk1-positive cells treated with Dox for indicated periods. Representative western blotting data from three independent experiments. **f** Effects of ICN1 induction on endothelial differentiation of iICN1-ECs. Flk1-positive cells treated with or without Dox for 3 days, and mRNA levels of ICN1, CD31, VE-cadherin, and Flk1 determined by qRT-PCR. mRNA level of each gene normalized to GAPDH, and data represent mean ± SD (*n* = 4). *p < 0.05, **p < 0.01, ***p < 0.001. bFGF basic fibroblast growth factor, BMP4 bone morphogenetic protein-4, Dox doxycycline, GAPDH glyceraldehyde 3-phosphate dehydrogenase, iICN1 inducible intracellular domain of Notch1, mESC mouse embryonic stem cell, PE phycoerythrin, VEGF vascular endothelial growth factor, DAPT  N-[N-(3, 5-difluorophenacetyl)-l-alanyl]-S-phenylglycine t-butyl ester

ESCs were differentiated into ECs according to the experimental timeline and differentiation conditions (Fig. 2a). To induce the formation of Flk1-positive mesodermal progenitor cells, culture medium containing leukemia inhibitory factor was removed from ESCs and the cells were cultured on type IV collagen-coated dishes and propagated in the presence of a complete medium containing bone morphogenetic protein-4, vascular endothelial growth factor, and basic fibroblast growth factor. After 5 days, Flk1-positive cells were isolated by magnetic-activated cell sorting and the endothelial phenotype was probed by FACS analysis. Double staining of the cells with anti-Flk1 and anti-CD31 antibodies exhibited that the sorted Flk1-positive cells also expressed CD31, an endothelial marker (Fig. 2b). To confirm the effect of Notch1 on the differentiation of ESCs into ECs, we evaluated the effect of Notch inhibition, via the γ-secretase inhibitor DAPT (*N*-(*N*-(3,5-difluorophenacetyl)-l-alanyl)-*S*-phenylglycine *t*-butyl ester) to block Notch1 endoproteolysis, on ESC-derived ECs. DAPT treatment markedly reduced the endogenous levels of ICN1 on day 2 and completely inhibited the production of ICN1 (Fig. 2c). Moreover, the expression levels of endothelial markers, CD31 and VE-cadherin, and Flk1 were also decreased in response to DAPT treatment, suggesting a crucial role of ICN1 in the endothelial differentiation of ESCs. To confirm these results, we next explored the effect of Dox-dependent induction of ICN1 in the endothelial differentiation of the Flk1-positive cells. The Flk1-positive cells were incubated with the endothelial differentiation medium in the absence or presence of Dox for 3 days. The cells exhibited cobblestone-shaped endothelial morphology and Dox treatment did not significantly affect the morphology of the cells (Fig. 2d). Dox treatment increased the protein level of ICN1 on day 1, and the protein levels of CD31 and VE-cadherin were also increased upon Dox treatment. The protein levels of VE-cadherin were significantly increased on day 2 after Dox treatment, whereas those of CD31 were markedly upregulated on day 3 (Fig. 2e). Moreover, qRT-PCR analysis showed that Dox-dependent ICN1 induction increased the mRNA levels of CD31, VE-cadherin, and Flk1 (Fig. 2f). Although the mRNA level of Flk1 was markedly augmented by Dox treatment, the protein level of Flk1 was not significantly affected by Dox treatment (Fig. 2e). Taken together, these results suggest that Notch1 activation is essential for the differentiation of Flk1-positive mesodermal progenitor cells to ECs.

### Notch1 promotes arterial specification of ESC-derived ECs

**Fig. 3:**
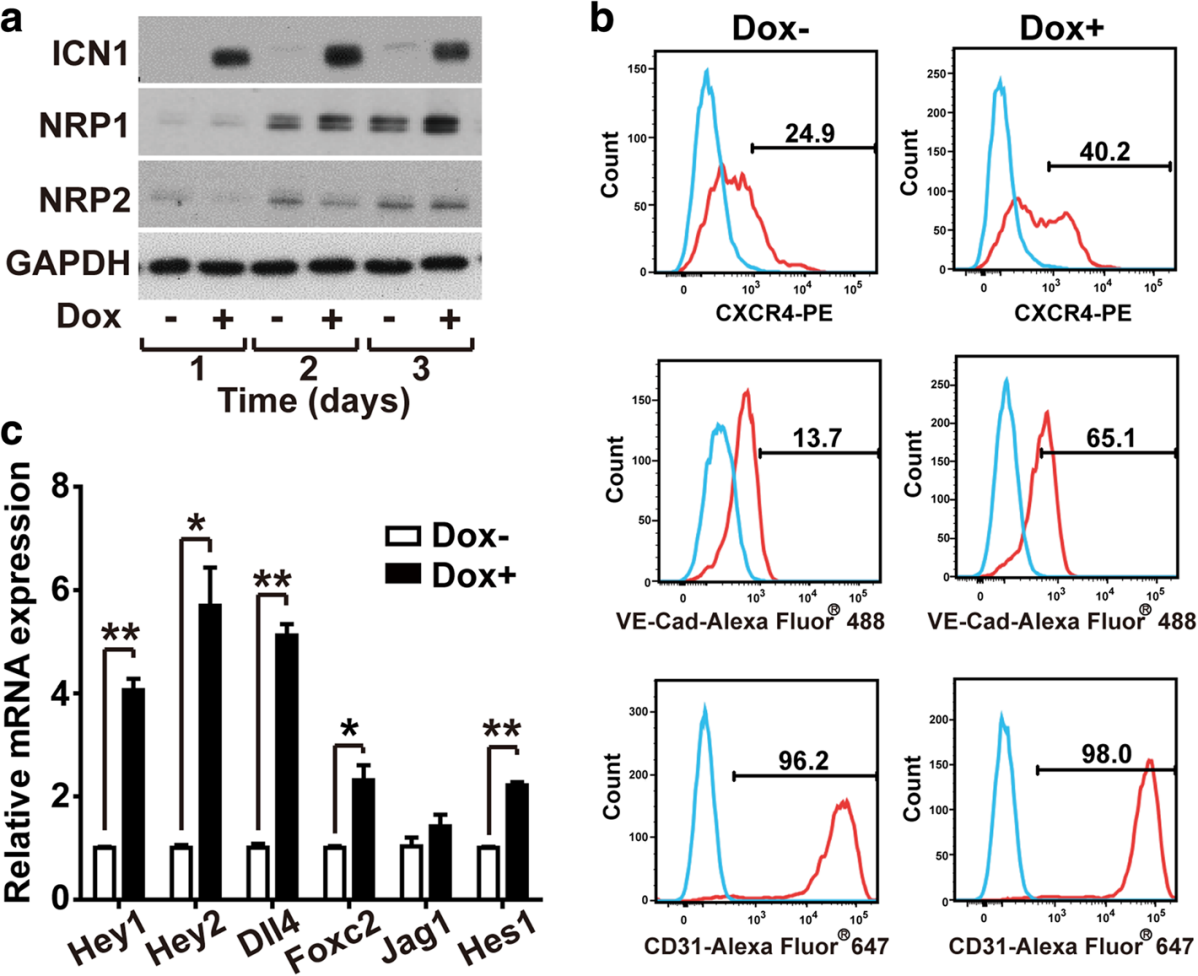
Effects of Notch1 activation in arterial specification of ESC-derived ECs. **a.** ICN1-induced expression of arterial EC markers. Protein levels of ICN1, NRP1 (arterial marker), NRP2 (vein marker), and GAPDH determined by western blotting analysis after Dox stimulation of iICN1-ECs for indicated time. **b** Effects of ICN1 induction on expression of EC surface markers. iICN1-ECs treated with or without Dox for 3 days, followed by FACS analysis after labeling with PE-conjugated anti-CXCR4, Alexa Fluor® 488-conjugated VE-cadherin, or Alexa Fluor® 647-conjugated CD31 antibodies. Percentages of the endothelial marker-positive populations indicated. **c** Effects of ICN1 induction on mRNA levels of Notch1 target genes in iICN1-ECs. mRNA levels of Hey1, Hey2, Hes1, Foxc2, Jag1, and Dll4 determined through qRT-PCR at day 1 after Dox treatment, and normalized GAPDH. Data represent mean ± SD (*n* = 3). *p < 0.05, **p < 0.01. Dox doxycycline, GAPDH glyceraldehyde 3-phosphate dehydrogenase, iICN1 inducible intracellular domain of Notch1, NRP neuropilin, PE phycoerythrin, VE-cad VE-cadherin

Notch signaling is essential for defining arterial ECs through inhibition of the venous lineage differentiation [17]. To explore whether Notch1 affects the specification of arterial and venous ECs, we examined the levels of arterial and venous markers in iICN1-ECs by western blotting analysis following Dox treatment. When iICN1-ECs were treated with Dox, the expression level of the arterial marker NRP1 increased, whereas the level of the venous endothelial marker NRP2 was not significantly altered (Fig. 3a). CXCR4, a chemokine receptor implicated in SDF-1α/CXCL12-mediated chemotaxis of endothelial progenitor cells [18], has been identified as an arterial endothelial marker [19]. Therefore, we next explored the effects of ICN1 induction on CXCR4 expression using FACS analysis. The expression of CXCR4 increased in response to Dox treatment in the Flk1-positive iICN1-ECs (Fig. 3b). Moreover, ICN1 induction significantly increased the expression of VE-cadherin. Most iICN1-ECs were positive for CD31 regardless of ICN1 induction, suggesting an ICN1-dependent increase of the arterial-type ECs expressing CXCR4, VE-cadherin, and CD31 in iICN1-ECs. Notch signaling has been reported to regulate the expression of Hey1 and Hey2 [20], Hes1 [21], Jagged1 [22], Dll4 [17], and Foxc2 [23] to stimulate arterial-type EC differentiation. To explore the effects of ICN1 induction on expression of Notch target genes, we quantified the mRNA levels of Notch target genes by qRT-PCR. As shown in Fig. 3c, Dox treatment significantly increased the mRNA levels of the Notch target genes, such as Hey1, Hey2, Dll4, Foxc2, and Hes1.

**Fig. 4:**
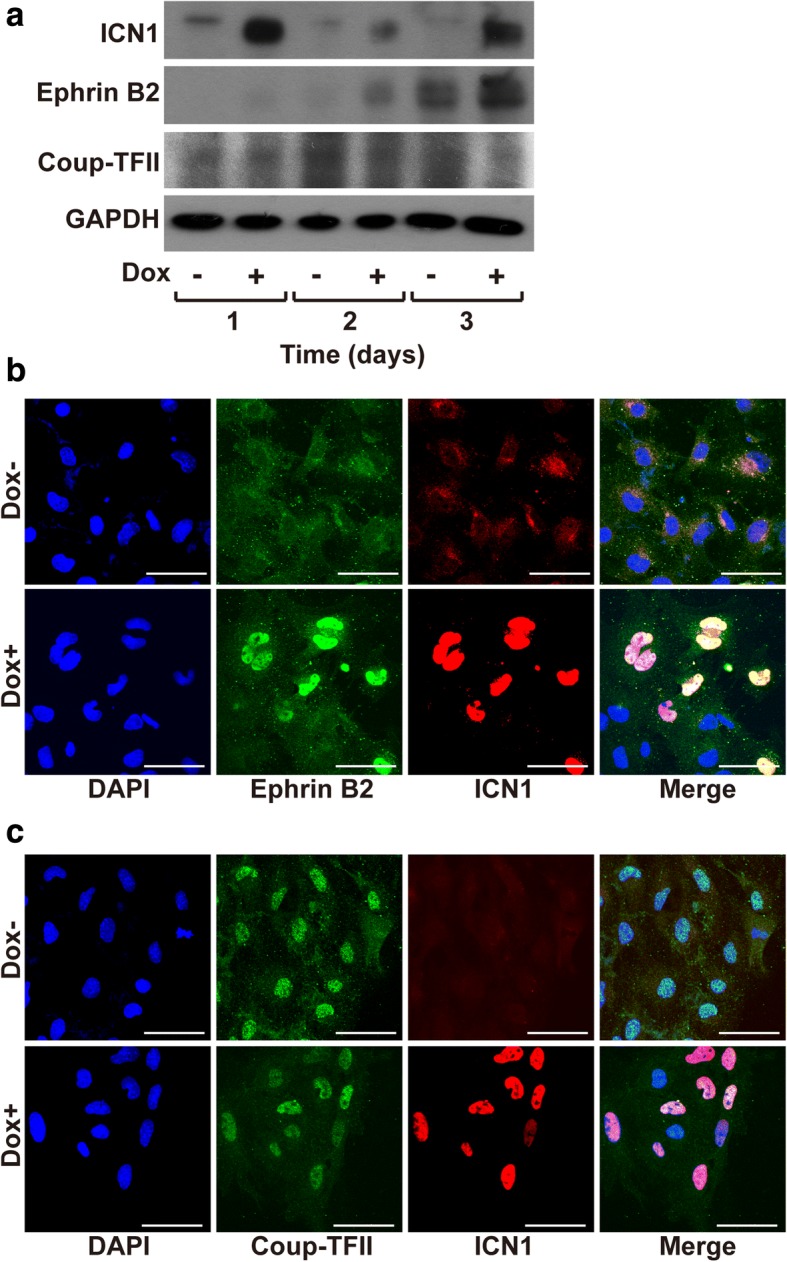
Effects of Notch1 activation on expression of arterial and venous EC markers in iICN1-ECs. **a** Western blotting of ICN1, arterial marker Ephrin B2, and venous marker Coup-TFII in iICN1-ECs treated with or without Dox for indicated time. **b, c** Immunostaining of Ephrin B2 and Coup-TFII in iICN1-ECs. Expression of Ephrin B2 (green), Coup-TFII (green), and ICN1 (red) probed by immunostaining in iICN1-ECs treated with or without Dox for 3 days. Nuclei stained with DAPI (blue), merged images of the three colors shown. Scale bars: 50 μm. DAPI 4′,6-diamidino-2-phenylindole, Dox doxycycline, GAPDH glyceraldehyde 3-phosphate dehydrogenase, iICN1 inducible intracellular domain of Notch1

We next examined the effects of ICN1 induction on the levels of Ephrin B2, an arterial EC-specific marker, and Coup-TFII, a venous EC-specific marker in the iICN1-ECs. Dox treatment increased the protein levels of Ephrin B2, whereas the protein levels of Coup-TFII were decreased (Fig. 4a). Moreover, Ephrin B2 expression was greatly upregulated in ICN1-positive cells (Fig. 4b). Although the decrease of Coup-TFII expression in ICN1-positive cells was not evident in the immunostaining results (Fig. 4c), these data suggest that Notch1 activation promotes arterial specification of ESC-derived ECs by increasing the expression of Notch target genes.

### Notch1 accelerates angiogenic activity of ESC-derived arterial ECs in vitro

**Fig. 5:**
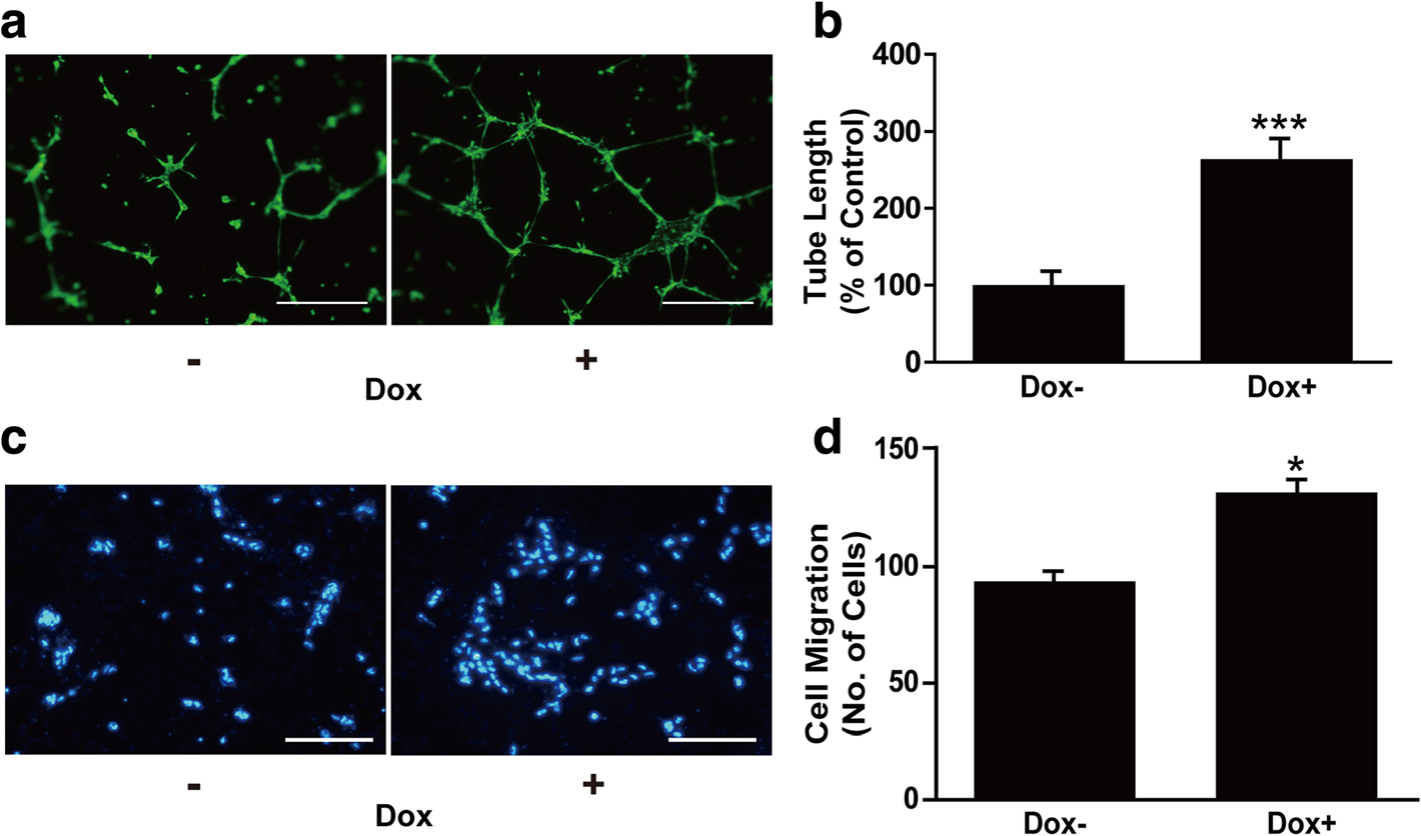
Effects of Notch1 activation on angiogenic properties of ESC-derived arterial ECs in vitro*.* **a** Effects of ICN1 induction on tube-forming ability of iICN1-ECs pretreated with (+) or without (–) Dox for 3 days. Tube networks stained with Calcein AM. Scale bars: 100 μm. **b** Quantification of data in (**a**). Data represent mean ± SD (*n* = 3). ***p < 0.001 vs Dox–. **c** Notch1-induced migration of iICN1-ECs. iICN1-ECs treated with or without Dox for 3 days, and migration abilities of the cells measured using Transwell migration assay in response to 10 ng/chemotactic ml VEGF. Scale bar: 50 μm. **d** Effects of ICN1 induction on EC migration quantified. Data represent mean ± SD (*n* = 9). *p < 0.05 vs Dox–. Dox doxycycline

Because ECs have angiogenic potential, including migration and tube formation for angiogenesis and tissue repair, we explored the impact of Notch1 signaling on the angiogenic activity of ESC-derived ECs. To determine the angiogenic potential of the cells, iICN1-ECs were seeded onto growth factor-reduced Matrigel in vitro. Tube formation was more persistent in Dox-treated iICN1-ECs compared to control cells (Fig. 5a, b). In a transwell migration assay, ICN1 induction significantly increased the migration of iICN1-ECs in response to VEGF treatment (Fig. 5c, d). These results indicate that Notch1 enhances the angiogenic function of ESC-derived arterial ECs.

### Notch1 activation stimulates therapeutic efficacy of ESC-derived arterial ECs in an ischemic hindlimb animal model

**Fig. 6:**
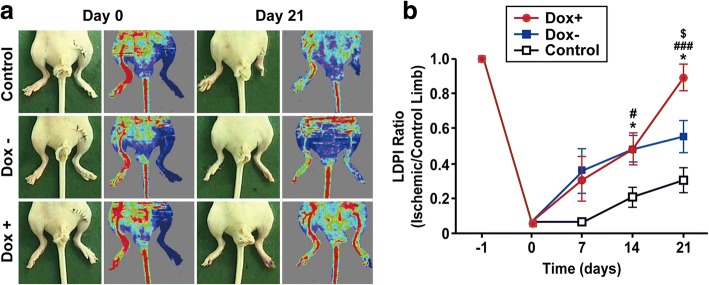
Effects of ICN1-induced arterial specification of ECs on vascular repair in ischemic limbs. **a** iICN1-ECs treated with or without Dox for 3 days and then injected intramuscularly to injury sites of nude mice with hindlimb ischemia. As negative control, HBSS buffer injected into ischemic limb. Representative photographs and laser Doppler perfusion imaging (LDPI) of mice on day 0 and day 21 shown. **b**. Time-course quantitative analysis of blood flow using LDPI of mice. Data represent mean ± SD (*n* = 4). *p < 0.05 Dox– vs control; #*p* < 0.05, ###*p* < 0.001 Dox+ vs control; $*p* < 0.05, Dox+ vs Dox–. Dox doxycycline

We evaluated whether Notch1 induction in ECs could contribute to the vascular repair of ischemic injuries. The femoral arteries of nude mice were ligated surgically to introduce ischemic injuries, and iICN1-ECs treated with or without Dox were injected intramuscularly into the site of ischemic injury. Mice were monitored for up to 21 days following iICN1-EC injection with the time-course measurement of blood flow. Analysis of mice on day 14 showed that injection of iICN1-ECs without Dox treatment significantly improved blood flow and limb salvage compared with control HBSS injection (Fig. 6a, b). Analysis of mice on day 21 showed that injection of iICN1-ECs with Dox treatment significantly improved blood flow and limb salvage compared to iICN1-ECs without Dox treatment. These results suggest that Notch1 strengthened the therapeutic efficacy of ESC-derived ECs in the treatment of hindlimb ischemia.

### Transplantation of Notch1-activated iICN1-ECs stimulates blood vessel formation in the ischemic hindlimb

**Fig. 7:**
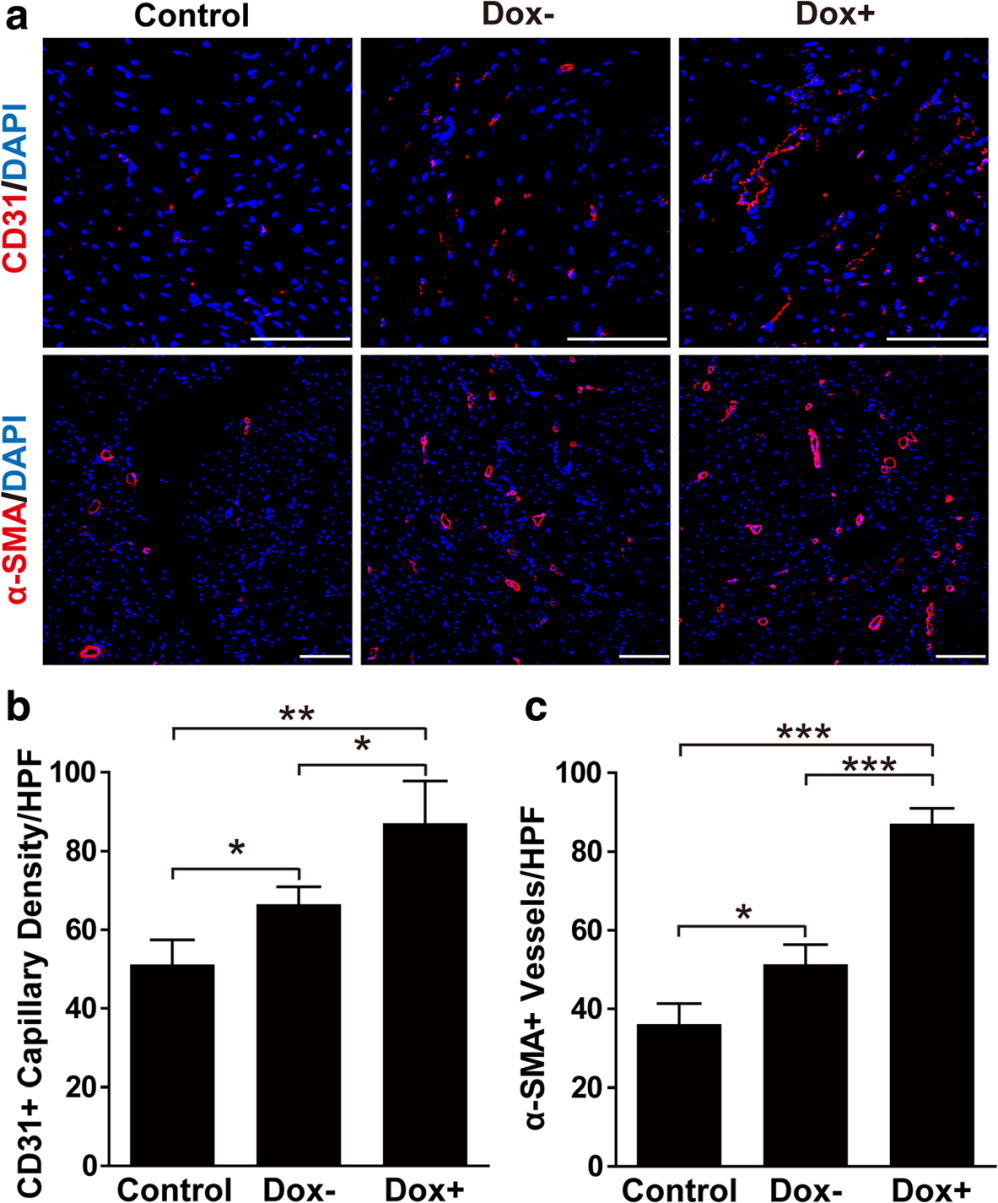
Effects of ICN1-induced arterial specification of ECs on therapeutic angiogenesis in vivo. **a** Representative images of ischemic hindlimb samples at 3 weeks after immunostaining with anti-CD31 antibody or anti-α-SMA antibody (red). Nuclei counterstained with DAPI (blue). **b, c** Quantitative analysis of CD31-positive capillaries (**b**) or α-SMA-positive blood vessels (**c**) in ischemic hindlimb on day 21. Average values presented as mean ± SD (*n* = 8). *p < 0.05, **p < 0.01, ***p < 0.001. Scale bar = 100 μm. α-SMA alpha smooth muscle actin, DAPI 4′,6-diamidino-2-phenylindole, Dox doxycycline, HPF high-power field

To explore the effects of Notch-activated iICN1-ECs on blood vessel formation, we measured the number of blood vessels, including CD31-positive endothelial capillaries and α-SMA-positive arterioles. iICN1-ECs without Dox treatment modestly increased the number of CD31-positive endothelial capillaries and α-SMA-positive arterioles in the murine ischemic hindlimb, whereas iICN1-ECs with Dox treatment prominently increased the number of CD31-positive and α-SMA-positive blood vessels compared with control groups (Fig. 7a–c). These results suggest that ICN1 induction in ECs promotes repair of hindlimb ischemia by increasing the number of blood vessels in vivo.

### Notch1 activation stimulates blood vessel formation mediated by iICN1-ECs in vivo

**Fig. 8:**
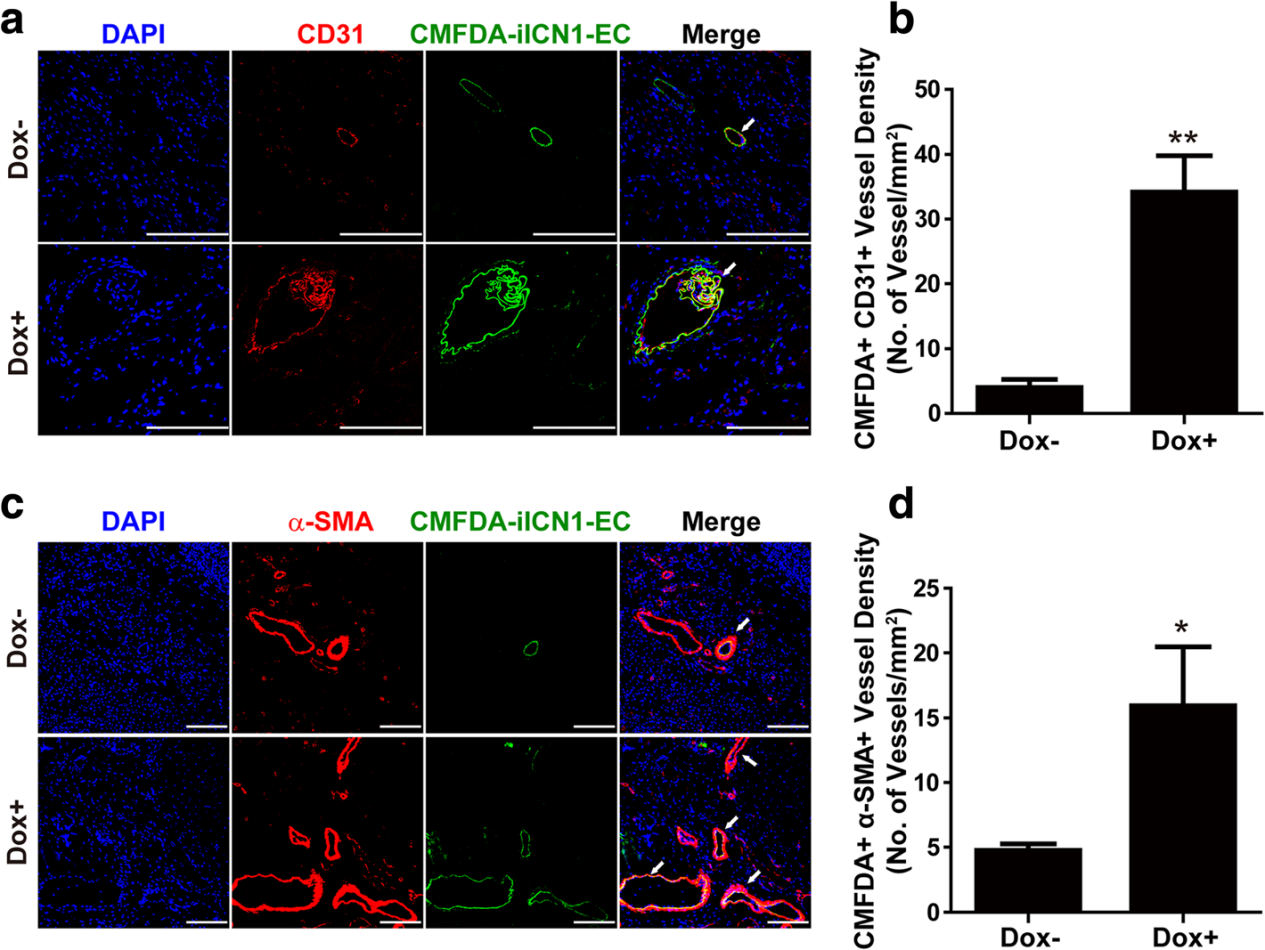
Effects of ICN1 induction on blood vessel formation mediated by ECs in vivo. iICN1-ECs treated with or without Dox for 3 days, labeled with CMFDA, and followed by transplantation into ischemic hindlimb. Hindlimb tissues on day 7 subjected to immunostaining with anti-CD31 antibody (**a**) and anti-α-SMA antibody (**c**) (red), nuclei stained with DAPI (blue). Arrows indicate α-SMA-positive or CD31-positive blood vessels containing CMFDA-positive iICN1-ECs. Scale bar = 100 μm. Numbers of capillaries containing both CMFDA and CD31 double-positive cells (**b**) and number of blood vessels bearing both CMFDA and α-SMA double-positive cells (**d**). Results presented as mean ± SD (*n* = 7). *p < 0.05, **p < 0.01. α-SMA alpha smooth muscle actin, DAPI 4′,6-diamidino-2-phenylindole, Dox doxycycline, EC endothelial cell, iICN1 inducible intracellular domain of Notch1

To determine whether transplanted iICN1-ECs stimulate angiogenesis by directly forming blood vessels, we injected CMFDA-labeled iICN1-ECs with or without Dox treatment into the ischemic hindlimb of mice. Ischemic hindlimb tissues were harvested 1 week following hindlimb ischemia surgery and sections of the ischemic tissues were stained with anti-CD31 and anti-α-SMA antibodies. We quantified the number of blood vessels containing exogenously transplanted CMFDA-iICN1-ECs. CMFDA/α-SMA and CMFDA/CD31 double-positive cells were detected in the CMFDA-iICN1-EC-transplanted ischemic hindlimb, and the numbers of CMFDA/CD31 and CMFDA/α-SMA double-positive blood vessels were increased by ICN1 induction (Fig. 8a–d). These results suggest that ICN1-induced arterial specification of ECs can stimulate the incorporation of transplanted ECs into newly formed blood vessels in the ischemic hindlimb.

## Discussion

ESCs may be useful for cell therapy as they have self-renewal and pluripotent differentiation potentials. Differentiation of ESCs into ECs is essential for the development of the embryonic vasculature. However, it remains difficult to induce the differentiation of ESCs toward arterial-type ECs. Notch1 signaling has been documented to regulate specification of arterial and venous ECs. It has been reported that Foxc1 and Foxc2 can regulate arterial specification by directly activating the Dll4 promoter [23]. In addition, Jagged1 [22] and Hes1 [21] have been reported to regulate arterial specification through regulation of Notch signaling. Shear stress-induced activation of Notch signaling has been reported to arrest the endothelial cell cycle in order to enable arterial specification [24]. Moreover, it has been reported that hypoxia induces differentiation of ESCs into arterial ECs through a HIF1α-dependent mechanism. HIF1α promoted differentiation of ESCs to arterial ECs through the expression of Etv2 and Notch1, and knockout of Notch1 gene abrogated the hypoxia-induced differentiation of ESCs to arterial ECs [25]. HIF1α activates not only Notch1 but also other hypoxia-induced signaling factors, and therefore it remains unclear whether Notch1 activation is sufficient for induction of arterial specification of ESC-derived ECs. In the present study, we demonstrated that induction of ICN1 led to increased expression of endothelial markers, including CD31 and VE-cadherin, in iICN1-ECs. Moreover, ICN1 induction elevated the levels of the arterial endothelial marker Nrp1, but not the venous type endothelial marker Nrp2, in the differentiated ECs. Therefore, these studies suggest that Notch1 is a key regulator of not only endothelial differentiation but also specification of arterial ECs.

Flk1-positive mesoderm angioblasts are reportedly involved in the differentiation of ECs [9]. Specifically, the specialization of mesodermal cells into mesodermal angioblasts is regulated by Etv2 (Er71) [26]. One study found that Etv2-knockout embryos died early and vasculature structure could not be detected during development [27]. Additionally, Etv2 can function downstream of Notch signaling to induce differentiation of Flk1^+^/PDGFRα^+^ primitive mesoderm into the vascular mesoderm [28]. Thus, Notch signaling is important for differentiation of mesoderm cells to mesoderm angioblasts. In the present study, we showed that ICN1 induction elevated the expression of CD31, VE-cadherin, and Nrp1 in Flk1-positive ECs, indicating specification of mesodermal progenitor cells to arterial ECs. Moreover, ICN1 induction promoted the specification of the hemogenic endothelium in differentiating cultures of mouse ESCs [14]. These results suggest that Notch1 activation regulates differentiation of ESCs not only to hematopoietic cells but also to ECs.

In the present study, we showed that Notch1 accelerated angiogenic potentials, including tube-forming and migration abilities, of ESC-derived ECs in vitro*.* Moreover, we demonstrated that Notch activation led to increased expression of CXCR4, which is implicated in the chemotaxis of angiogenic progenitor cells to ischemic tissues [18]. The CXCL12/CXCR4 signaling axis plays a critical role in coronary artery development [29, 30]. During development, CXCL12 drives migration of CXCR4-positive cells, including ECs, and the CXCL12/CXCR4 signaling axis plays a significant role in angiogenesis in various organs [31]. Notch signaling has been shown to regulate CXCR4 expression and the migration of mesenchymal stem cells [32]. Induction of Notch1 in iICN1-ECs directly formed blood vessel due to enhanced angiogenic properties. Consistent with these findings, CXCL12/CXCR4 signaling was found to enhance capillary-like tube formation of human ESC-derived ECs in vitro [33]. Taken together, these results suggest that Notch1 activation promotes the angiogenic potentials of ESC-derived ECs through upregulation of the CXCL12/CXCR4 signaling axis.

Despite previous studies suggesting that Notch1 is important for vascular development, it has not been reported that arterial-type ECs are more effective for vascular repair in the ischemic hindlimb model. We demonstrated here that the therapeutic potential of arterial-type ECs was more potent than control ECs in an ischemic hindlimb animal model. Transplantation of ICN1-induced arterial ECs more potently stimulated blood perfusion through the formation of new blood vessels than control cells. One study reported that Flk1^+^/VE-cadherin^+^ ECs derived from induced pluripotent stem cells organized into well-developed vascular structures in vitro and incorporated into CD31^+^ neovessels in Matrigel plugs implanted in nude mice in vivo [16]. Recently, it was demonstrated that arterial ECs differentiated from human pluripotent stem cells were able to significantly enhance survival rates compared with venous endothelial cells in a mouse myocardial infarction model [34]. These results suggest that Notch1-induced arterial-type ECs may be useful in the treatment of ischemic diseases, although the mechanisms implicated in the arterial EC-mediated therapy of ischemic diseases as well as the effects of enhanced vasculogenesis require further clarification.

## Conclusions

The present study demonstrates that induced expression of ICN1 stimulated specification of iICN1-ECs to arterial ECs and increased expression of CXCR4. Moreover, induced expression of ICN1 increased endothelial tube formation and migration of the iICN1-ECs in vitro. Intramuscular transplantation of Notch1-induced arterial ECs effectively restored the blood flow in the ischemic hindlimb mouse model and augmented vasculogenesis in the ischemic limbs. Taken together, our findings contribute to understanding how modulation of Notch signaling affects ESC-derived ECs for arterial specification and their therapeutic efficacy.

## Additional file

Additional file 1: **Table S1.** Mouse primer sequences used in qRT-PCR. (DOCX 18 kb)

## Abbreviations

Dox: Doxycycline
EC: Endothelial cell
ESC: Embryonic stem cell
FACS: Fluorescence-activated cell sorter
HBSS: Hank’s Balanced Salt Solution
iICN1: Inducible intracellular domain of Notch1
LDPI: Laser Doppler perfusion imaging
PE: Phycoerythrin
qRT-PCR: Real-time polymerase chain reaction
