# Supplementary material for: Role of Notch1 in the arterial specification and angiogenic potential of mouse embryonic stem cell-derived endothelial cells
Source: Stem Cell Res Ther. 2018 Jul 18;9:197. doi: 10.1186/s13287-018-0945-7 (PMC6052544; doi:10.1186/s13287-018-0945-7)
Supplement: Supplementary file 1 — Table S1. Mouse primer sequences used in qRT-PCR. (DOCX 18 kb) [file 13287_2018_945_MOESM1_ESM.docx]

**Additional file 1**

**Table S1. Mouse primer sequences used in qRT-PCR**

| Genes | Primer Sequences | |
| --- | --- | --- |
|  | Forward | Reverse |
| Hey1 | CTTGCAGATGACCGTGGA | GTGAGGCATTCCCGAAAC |
| Hey2 | TGAAGATGCTCCAGGCTACA | CACTCTCGGAATCCAATGCT |
| Dll4 | TGTCTCCACGCCGGTATTG | AGGTCGTCTCCCGGTGTGT |
| Jag1 | GCCGAGGTCCTACACTTTGCT | GTGGGCAATCCCTGTGTTTT |
| Hes1 | ACCCCAGCCAGTGTCAACA | TGTGCTCAGAGGCCGTCTT |
| VE-cadherin | CGCCAAAAGAGAGACTGGAT | CGTTGGACTTGATCTTTCCC |
| Flk1 | GCTTTCGGTAGTGGGATGAA | TTGGTGAGGATGACCGTGTA |
| GAPDH | ATGAATACGGCTACAGCAACAGG | CTCTTGCTCAGTGTCCTTGCTG |
| Foxc2 | ACAACCTGTCACTCAATG | CTTAACCACGACTTTCTTCT |
| ICN1 | CTGGCTTGTGGTAGCAAGGA | CCGTAGTGGGTTGTACTGGC |
| CD31 | CTGGTGCTCTATGCAAGCCT | AGTTGCTGCCCATTCATCAC |
